# Mapping Intellectual Structure for the Long Non-Coding RNA in Hepatocellular Carcinoma Development Research

**DOI:** 10.3389/fgene.2021.771810

**Published:** 2022-01-03

**Authors:** Zhifeng Lin, Xiaohui Ji, Nana Tian, Yu Gan, Li Ke

**Affiliations:** ^1^ Key Laboratory for Major Obstetric Diseases of Guangdong Province, Department of Medical Record, The Third Affiliated Hospital of Guangzhou Medical University, Guangzhou, China; ^2^ Department of Obstetrics and Gynaecology, Sun Yat-Sen Memorial Hospital, Sun Yat-Sen University, Guangzhou, China; ^3^ Department of Medical Record, The Fifth Affiliated Hospital of Guangzhou Medical University, Guangzhou, China

**Keywords:** lncRNA, hepatocellular carcinoma, bibliometric, citespace, database

## Abstract

**Background:** Emerging research suggests that long non-coding RNAs (lncRNAs) play an important role in a variety of developmental or physiological processes of hepatocellular carcinoma (HCC). Various differentially expressed lncRNAs have been identified in HCC. Thus, a deeper analysis of recent research concerning lncRNA and HCC development could provide scientists with a valuable reference for future studies.

**Methods:** Related publications were retrieved from the Web of Science Core Collection database. CiteSpace version 5.6.R4 was employed to conduct bibliometric analysis. Several network maps were constructed to evaluate the collaborations between different countries, institutions, authors, journals, and keywords.

**Results:** A total of 2,667 records were initially found from the year of 2010–2020. The annual related publications output had increased dramatically during these years. Although China was the most prolific country in terms of research publication, the United States played a leading role in collaborative network. The Nanjing Medical University was the most productive institute in the field of lncRNAs in HCC development. Gang Chen was the most prolific researcher, while Yang F was the most frequently co-cited author. Oncotarget, Cell, and Oncogene were the most highly co-cited journals. The most recent burst keywords were interaction, database, and pathway.

**Conclusion:** This study provides a comprehensive overview for the field of lncRNAs in HCC development based on bibliometric and visualized methods. The results would provide a reference for scholars focusing on this field.

## Introduction

Liver cancer is expected to be the sixth most commonly diagnosed cancer, and it ranks as the fourth leading cause of cancer death. With the absence of early diagnosis and limited treatment methods, Bray et al. estimates the global incidence of liver cancer to be 841,000 new cases and 782,000 deaths in 2018, with greater prevalence in Northern and Western Africa and Eastern and South-Eastern Asia (Bray et al., 2018). Hepatocellular carcinoma (HCC) is the most prevalent form of primary liver cancer, which accounts for >90% of primary liver cancer cases (Llovet et al., 2021). The primary risk factors for HCC include chronic viral hepatitis infection (hepatitis B or C), aflatoxin B1 intake, obesity, and excessive alcohol consumption (Chhonker et al., 2021; Choi et al., 2021; Guan et al., 2021; Hwang et al., 2021; Lockart et al., 2021). These factors may be responsible for cancer-related mutations, DNA damage, and epigenetic alterations, resulting in inactivated oncogenes or inactivated tumor suppressor genes, ultimately leading to HCC development (Rovida et al., 2015; Ally et al., 2017; Dang et al., 2017). Current treatment strategies for HCC include specific kinase inhibitors, anti-hepatitis vaccine, and liver resection and transplantation (Dageforde et al., 2021; Pattyn et al., 2021; Sbenati et al., 2021; Yamamoto et al., 2021). These therapeutic approaches in combination with biomarker screening reduce HCC-related death. However, its treatment efficiency is far from satisfactory. Thus, novel treatment approaches with higher efficacy for HCC are an urgently needed.

Earlier studies related to HCC primarily concentrated on the coding genes in identified regions with their pivotal roles in many central biological processes (Ladero et al., 1996; Thorgeirsson et al., 2006; Park et al., 2016). However, only 2% of human genome transcripts comprise protein coding sequences, and nearly 98% of sequences have no coding for proteins; the transcripts that cannot be ultimately translated into proteins have been characterized as non-coding RNAs (ncRNAs) (Rosenbloom et al., 2012; Beermann et al., 2016). There is a growing body of evidences that support that these evolutionarily conserved ncRNAs, particularly in the long non-coding RNAs (lncRNAs), which are more than 200 nucleotides in length, play a regulatory role in various developmental or physiological processes of HCC (Huang et al., 2020; Qin et al., 2020; Zuo et al., 2020). The number of transcribed lncRNAs continues to grow rapidly (Xie et al., 2021). Several studies have proved that these lncRNAs are differentially expressed in a variety of tissues and HCC cells (Hongfeng et al., 2020; Topel et al., 2020; Gamaev et al., 2021). Currently, the function of a small fraction of lncRNAs in HCC has been clearly defined, such as DNA binding (Zhou et al., 2020), associating with proteins (Gou et al., 2018), RNA interaction (Zhou and Xia, 2020), and producing small peptides (Wu et al., 2020). Intriguingly, some HCC-related lncRNAs have been founded in bodily fluids, making them attractive alternative biomarkers for HCC (Burenina et al., 2021). Studies have shown that these lncRNAs serve as potential cancer biomarkers and therapeutic targets for treating HCC (Klingenberg et al., 2017; Ghafouri-Fard et al., 2020; Matboli et al., 2020; Sheng et al., 2021).

Recently, there has been increased interest in bibliometric studies. For example, Li et al. explored the research hotspots of external beam radiotherapy in prostate cancer (Li R. et al., 2021); the hotspots of the role of anesthesia on tumor prognosis was performed by Luo et al. (2021); Martynov et al. conducted a bibliometric study to find the hotspot trend related to neuroblastoma research (Martynov et al., 2020). However, few studies are available for the research hotspots of lncRNAs in HCC development by using bibliometric methods. Bibliometric analysis regarding the application of lncRNAs in HCC research can provide more detailed insights into how lncRNAs play a critical role in HCC development. In recent years, bibliometric approach has been used as the method for quantitative and qualitative analysis (Li Q. et al., 2021). Researchers may have a better understanding of research trends and research hotspots in particular areas from bibliometric analysis (Carollo et al., 2021; Kilicaslan et al., 2021). Thus, we preferentially used CiteSpace to explore the frontiers of the roles of lncRNAs in HCC development. The results would provide scientists with a valuable reference for future studies.

## Materials and Methods

All relevant publications were retrieved from the Web of Science Core Collection (WoSCC) database with the following search strategies: TS = (Long non coding RNA OR LncRNA OR Lnc RNA OR Long noncoding RNA) AND TS = (Hepatocellular carcinoma OR Hepatocarcinoma OR HCC patient OR HCC cancer OR Hepatic carcinoma) AND Document types = (Article OR Review) AND Language = English, with a limited time frame from 2010 to 2020, index = Science Citation Index Expanded (SCI-EXPANDED). The data extraction was completed for all included studies within the day on August 20, 2021. In total, 2,667 publications were finally included. The data were retrieved by one author (ZL) and double checked by another author (NT). We then cleaned the data, such as merging “TAIWAN” into “China” in country cooperative analysis, unifying “long noncoding rna” and “long non-coding rna” as “LncRNA” in keyword co-occurrence analysis, and so on.

The CiteSpace version 5.6.R4 was used to conduct bibliometric analyses (Chen, 2006). Some valuable parameters were included, such as publication number, total number of citations, impact factor (IF), citation burst, and centrality. Productivity was calculated for individuals, countries, and institutions on the basis of the total number of publications. The total number of citations was used to evaluate the international impact on both authors and articles (Chen et al., 2020). IF was used for assessing the international impact of journals, and it was obtained from the 2020 Journal Citation Reports (Pacey, 2020). Burst keywords referred to keywords which were cited frequently over a period of time, and they were considered indicators of research frontiers (Zhu et al., 2020). Centrality was a critical parameter for measuring the importance of nodes in a network. The nodes with a higher centrality (≥0.1) in the network were highlighted using purple rings and were usually regarded as high influence in a network (Liang et al., 2017).

The retrieved data were used for bibliometric analysis, and analyses were performed with the software CiteSpace version 5.6.R4, such as cooperative analysis, document co-citation analysis, keyword co-occurrence, timeline view of keywords, and citation burst analysis of keywords. The top 50 most cited articles in each time splicing with 1 year were selected to create a network. In order to highlight the key network, minimum spanning tree was used to explore the co-author citation. Minimum duration was set to 1 in the burst analysis of keywords.

## Results

### Publication Outputs

**FIGURE 1:**
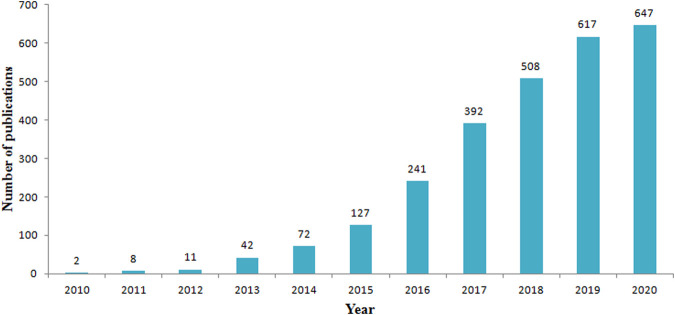
The number of annual publications on the roles of lncRNAs in HCC development research from 2010 to 2020.

A total of 2,667 records were initially found based on the search criteria. Figure 1 shows the distribution of publications related to the field of lncRNAs in HCC development from year 2010 to year 2020. The literature showed an overall rising trend in the total number of scientific publications from 2 in 2010 to 647 in 2020. Remarkably, the annual output of papers was nearly doubled compared to the previous year after 2014. It indicated that the annual related publications output had increased dramatically during year 2014 to year 2020.

### Distribution by Countries and Institutions

**FIGURE 2:**
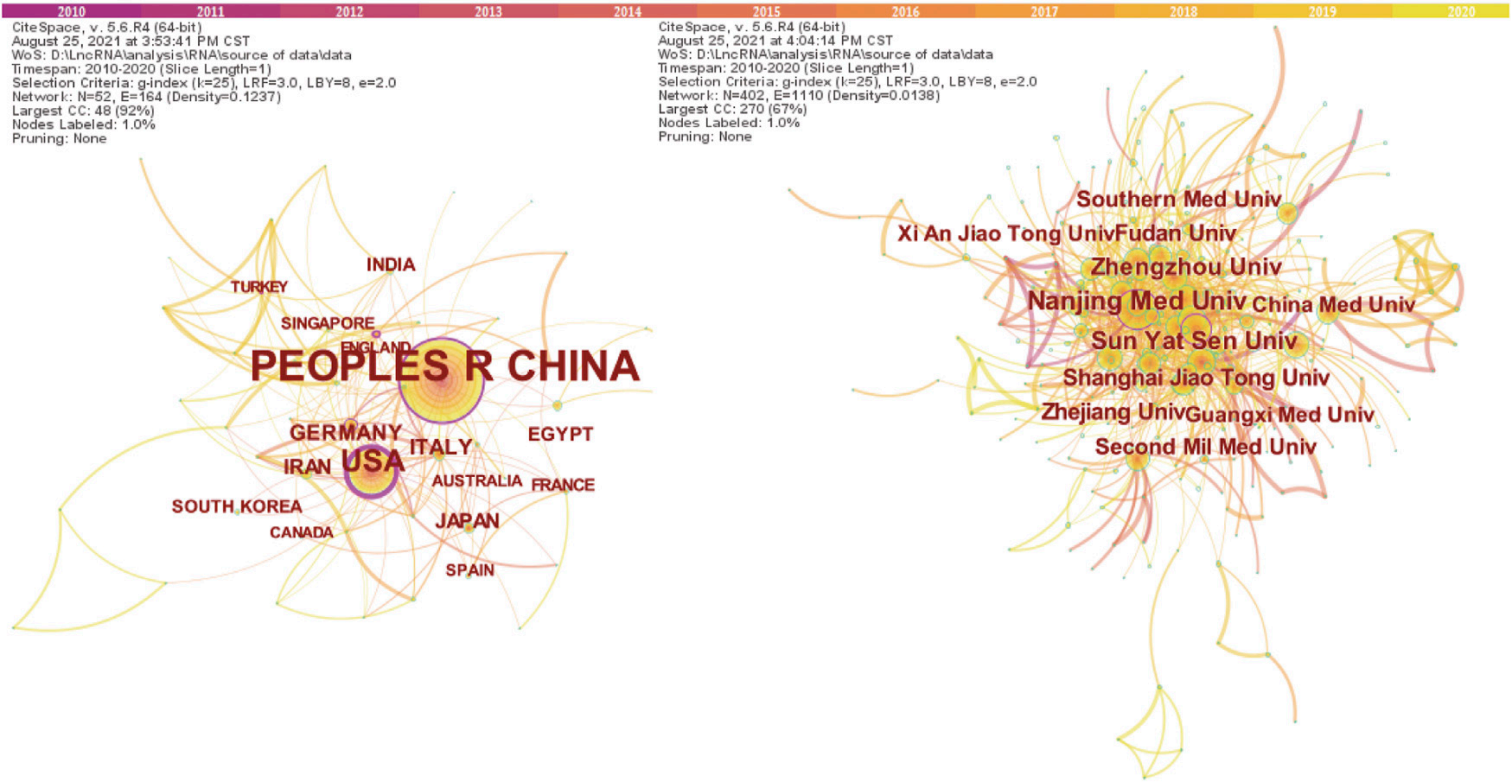
The co-occurrence map of countries **(A)** (T ≥ 13) and institutions **(B)** (T ≥ 62) in the field of lncRNAs in HCC development research.

There were 52 different countries which were identified in the network between year 2010 and year 2020. The country with at least 13 publications (T ≥ 13) are shown in Figure 2A. We computed the centrality for each node in order to identify strongly influential nodes in a network; the node with purple circle was considered more influential in the network. The centrality value for each country is shown in Table 1. The United States had an advantage in highest centrality (0.60), followed by China (0.39) and Germany (0.12). The top 10 countries which produced scientific publications concerning lncRNAs and HCC development are also presented in Table 1; the results indicated that China was the first leading country regarding the amount of publications (2,292), followed by the United States (231), Italy (39), and Germany (38). Among all the countries, China and the United States played a leading role in collaborative networks.

**TABLE 1:** The top 10 countries and institutions involved in the roles of lncRNAs in HCC development research.

Rank	Country	Count	Centrality	Institution	Country	Count	Centrality
1	China	2,292	0.39	Nanjing Med Univ	China	158	0.10
2	United States	231	0.60	Sun Yat Sen Univ	China	112	0.13
3	Italy	39	0.06	Zhengzhou Univ	China	100	0.06
4	Germany	38	0.12	Shanghai Jiao Tong Univ	China	87	0.05
5	Iran	35	0.03	Zhejiang Univ	China	86	0.08
6	Japan	34	0.01	Fudan Univ	China	80	0.09
7	Egypt	25	0.06	Second Mil Med Univ	China	68	0.08
8	India	22	0.03	Southern Med Univ	China	66	0.03
9	South Korea	19	0.00	Guangxi Med Univ	China	62	0.03
10	France	17	0.02	Xi An Jiao Tong Univ	China	62	0.04

**FIGURE 3:**
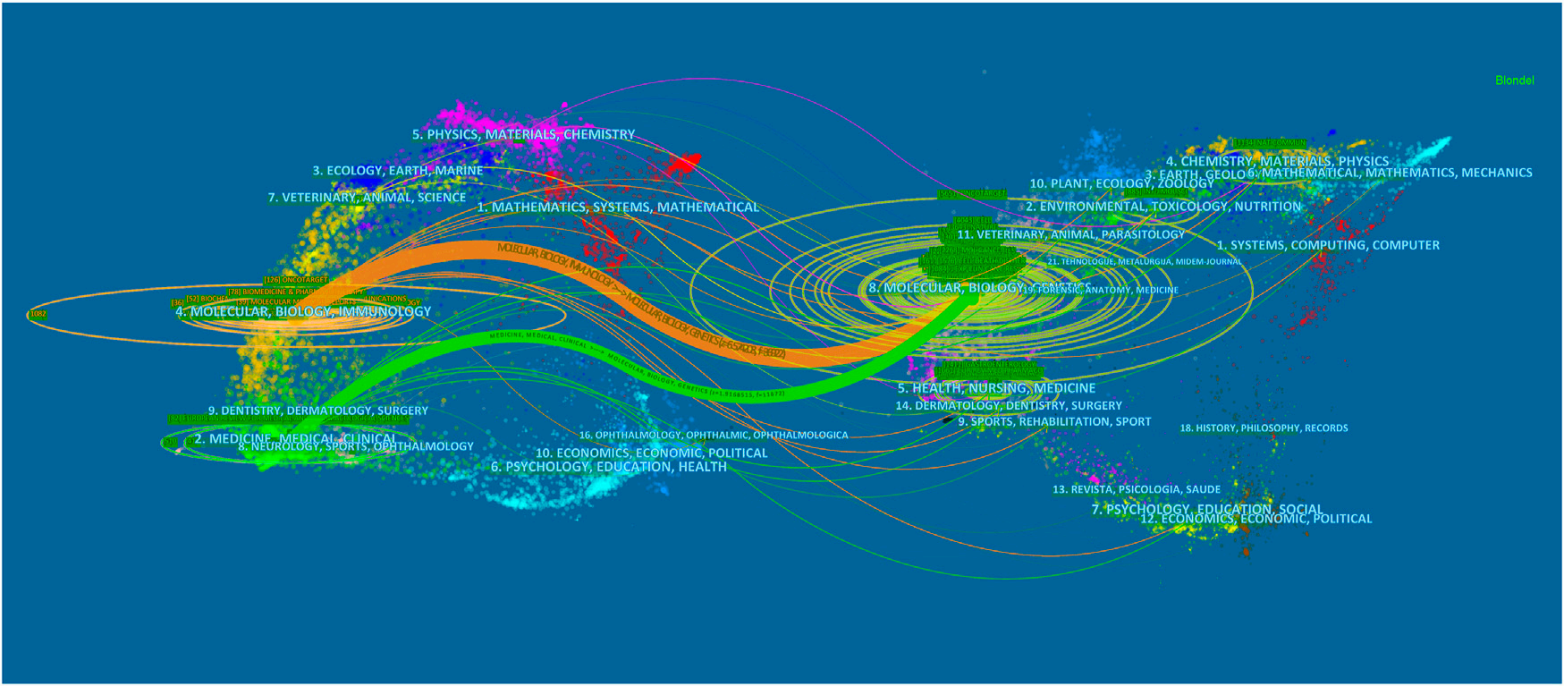
The dual-map overlay of journals related to lncRNA in HCC research. Notes: The citing journals are on the left, the cited journals are on the right, and the colored path represents citation relationship.

Among the major international institutions, all of the top 10 contributing institutions were from China (Table 1), including the Nanjing Medical University (*n* = 158), Sun Yat Sen University (*n* = 112), and Zhengzhou University (*n* = 100). Institutions with centrality ≥0.1 included Nanjing Medical University and Sun Yat Sen University, suggesting that these institutions had an important role in this field. As Figure 3B (T ≥ 62) shows, the three aforementioned institutions were in a unique position in a collaborations network.

### Distribution by Journals and Co-Cited Journals

The dual-map overlay of journals stands for the topic distribution of academic journals (Chen and Leydesdorff, 2014) (Figure 3). The citing journals are located on the left, while the cited journals are on the right, and the colored paths indicate the citation relationships. There were two citation paths. The first orange path was for papers published in molecular/biology/genetics journals, which were mainly cited by the studies published in molecular/biology/immunology journals; the next green path was for papers published in molecular/biology/immunology journals, which were mainly cited by the studies published in medicine/medical/clinical area.

The top 10 co-cited journals were identified with highly frequent citations (Table 2). Oncotarget, one of the top 10 journals, had the most co-citations (1,751 citations, IF = none), followed by Cell (1,570 citations, IF = 41.582), Oncogene (1,460 citations, IF = 9.867), and Cancer Research (1,442 citations, IF = 12.701). Among these top 10 journals, it was noteworthy that the top six journals came from the United States, and eight of them were at the Q1 JCR division.

**TABLE 2:** The top 10 co-cited journals involved in the roles of lncRNAs in HCC development research.

Rank	Journal title	Country	IF (2020)	JCR division	Total number of citations
1	Oncotarget	United States	—	—	1,751
2	Cell	United States	41.582	Q1	1,570
3	Oncogene	United Kingdom	9.867	Q1	1,460
4	Cancer Research	United States	12.701	Q1	1,442
5	Nature	United Kingdom	49.962	Q1	1,403
6	Hepatology	United States	17.425	Q1	1,354
7	Plos One	United States	3.24	Q2	1,318
8	Molecular Cancer	United Kingdom	27.401	Q1	1,232
9	Cancer Letters	Netherlands	8.679	Q1	1,222
10	CA-Cancer J. Clin	United States	508.702	Q1	1,118

### Distribution by Authors and Co-Cited Authors

**FIGURE 4:**
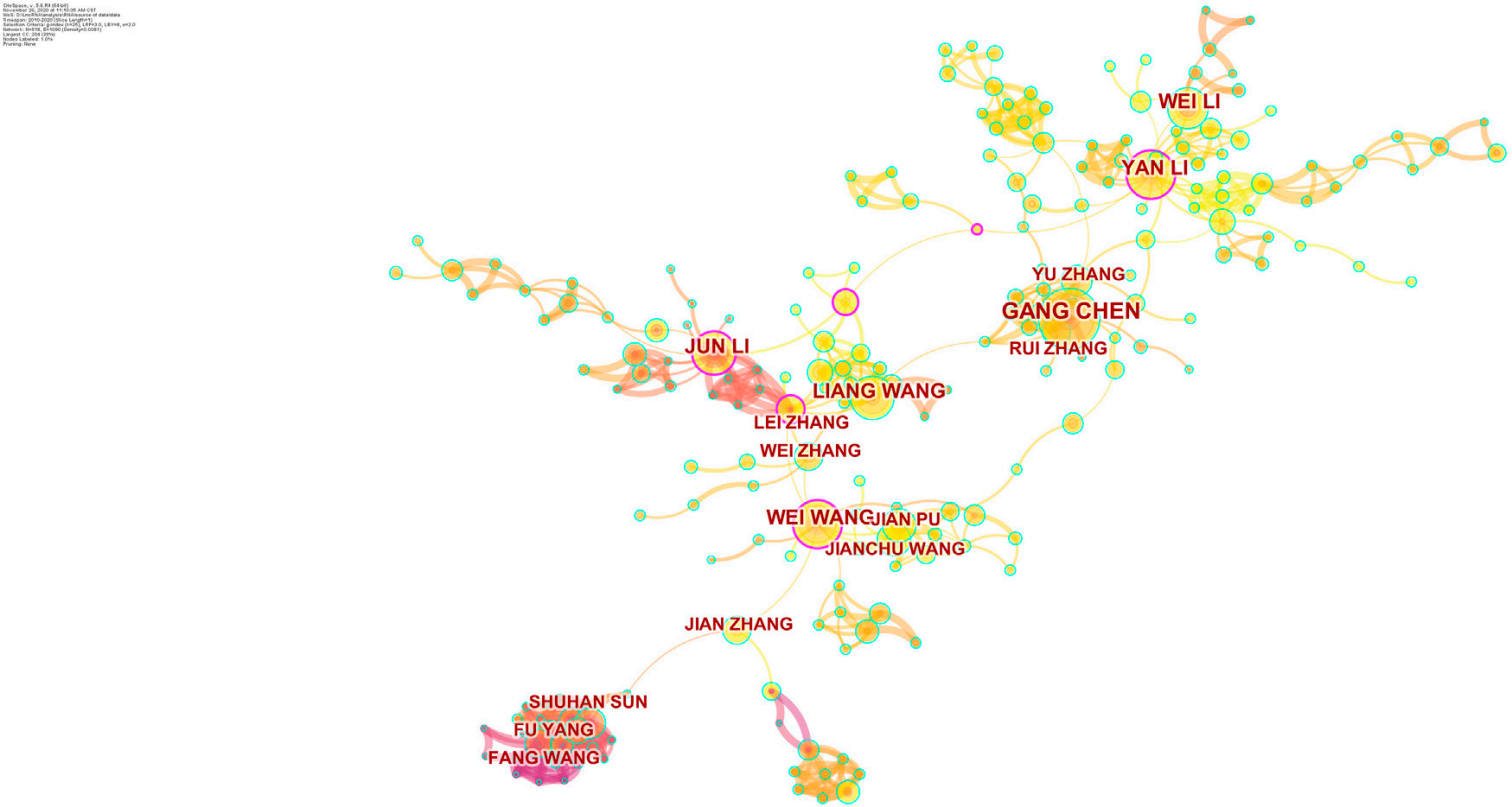
Network map of authors engaged in the roles of lncRNAs in HCC development research.

**FIGURE 5:**
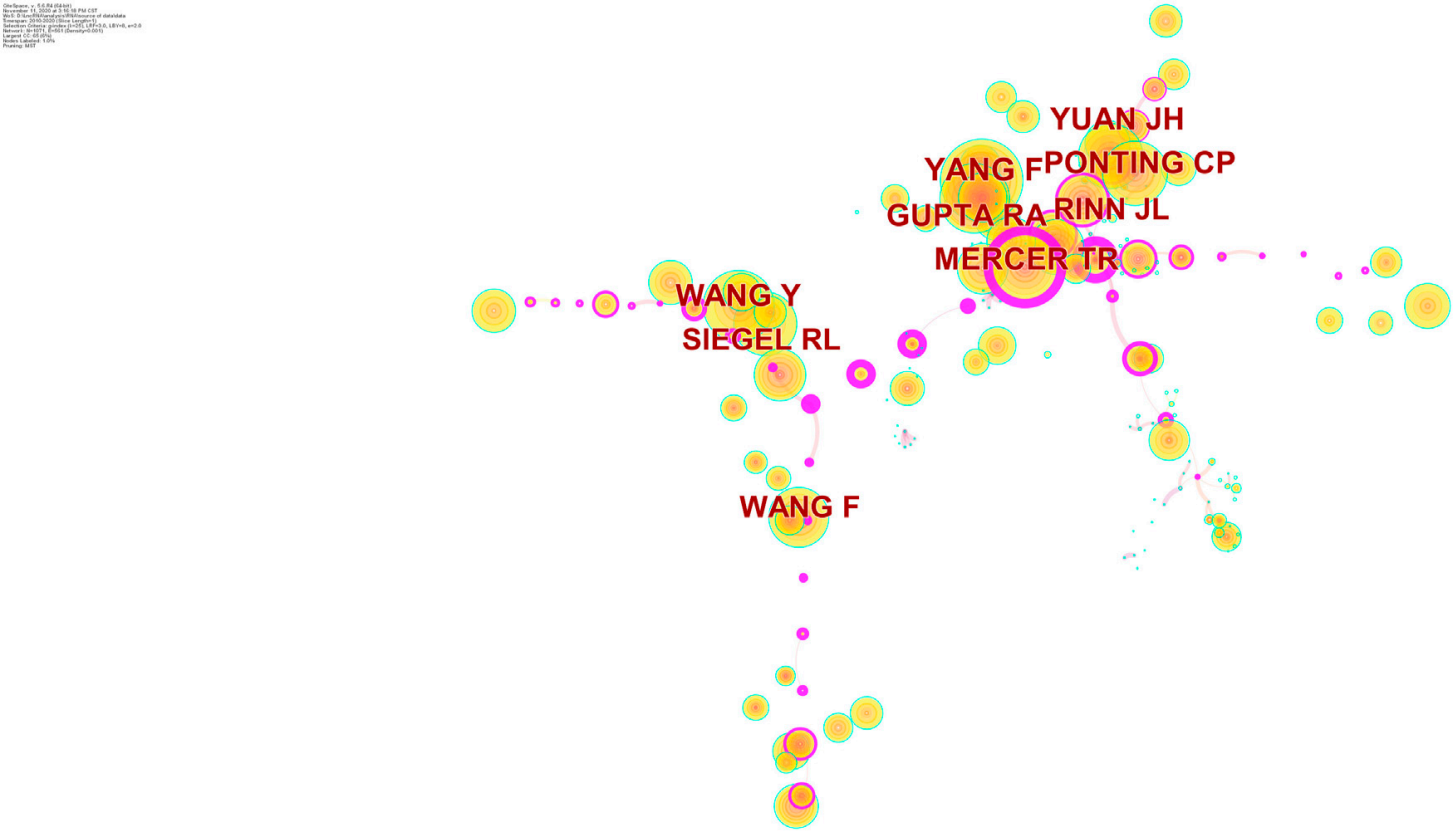
Network map of cited authors engaged in the roles of lncRNAs in HCC development research.

Figure 4 shows the authors who are distributed in the cluster with the largest size. Cooperation relationships were indicated by directed edges among nodes. When the relationship between the two authors was stronger, the line could be also thicker. Table 3 shows the top 10 prolific researchers. The number of published papers varied from 11 to 23 for different authors. Gang Chen published the largest amount of articles (23 publications), followed by Wei Wang (18 publications), and Yan Li (18 publications). Remarkably, only one of the top 10 prolific authors was included in the top 10 co-cited authors. The authors with at least 300 co-citations (T ≥ 300) were used to make the network (Figure 5). This type of knowledge map could clearly show the co-cited authors with high frequency. According to Figure 5, Yang F and Wang Y had the largest circles.

**TABLE 3:** The top 10 authors and co-cited authors that published articles on the roles of lncRNAs in HCC development research.

Rank	Author	Count	Co-cited author	Co-citation
1	Gang Chen	23	Yang F	451
2	Wei Wang	18	Wang Y	395
3	Yan Li	18	Gupta RA	391
4	Li Liu	17	Ponting CP	363
5	Jun Li	16	Mercer TR	361
6	Liang Wang	16	Yuan JH	358
7	Wei Li	15	Wang F	341
8	Jianchu Wang	12	Siegel RL	341
9	Jian Pu	12	Li J	338
10	Hua Tang	11	Rinn JL	303

### Analysis of Keywords

**FIGURE 6:**
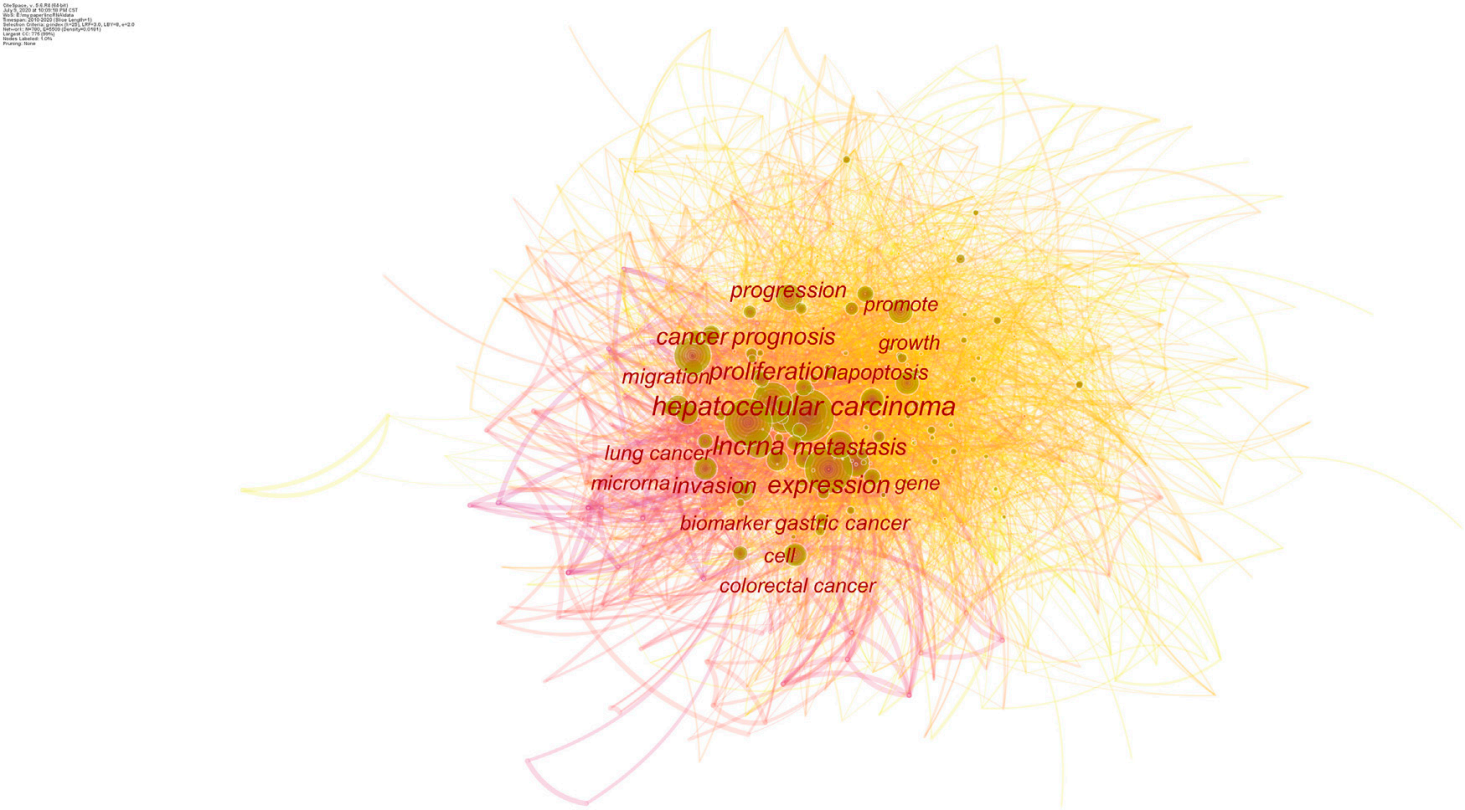
Network map of keywords engaged in the roles of lncRNAs in HCC development research.

**FIGURE 7:**
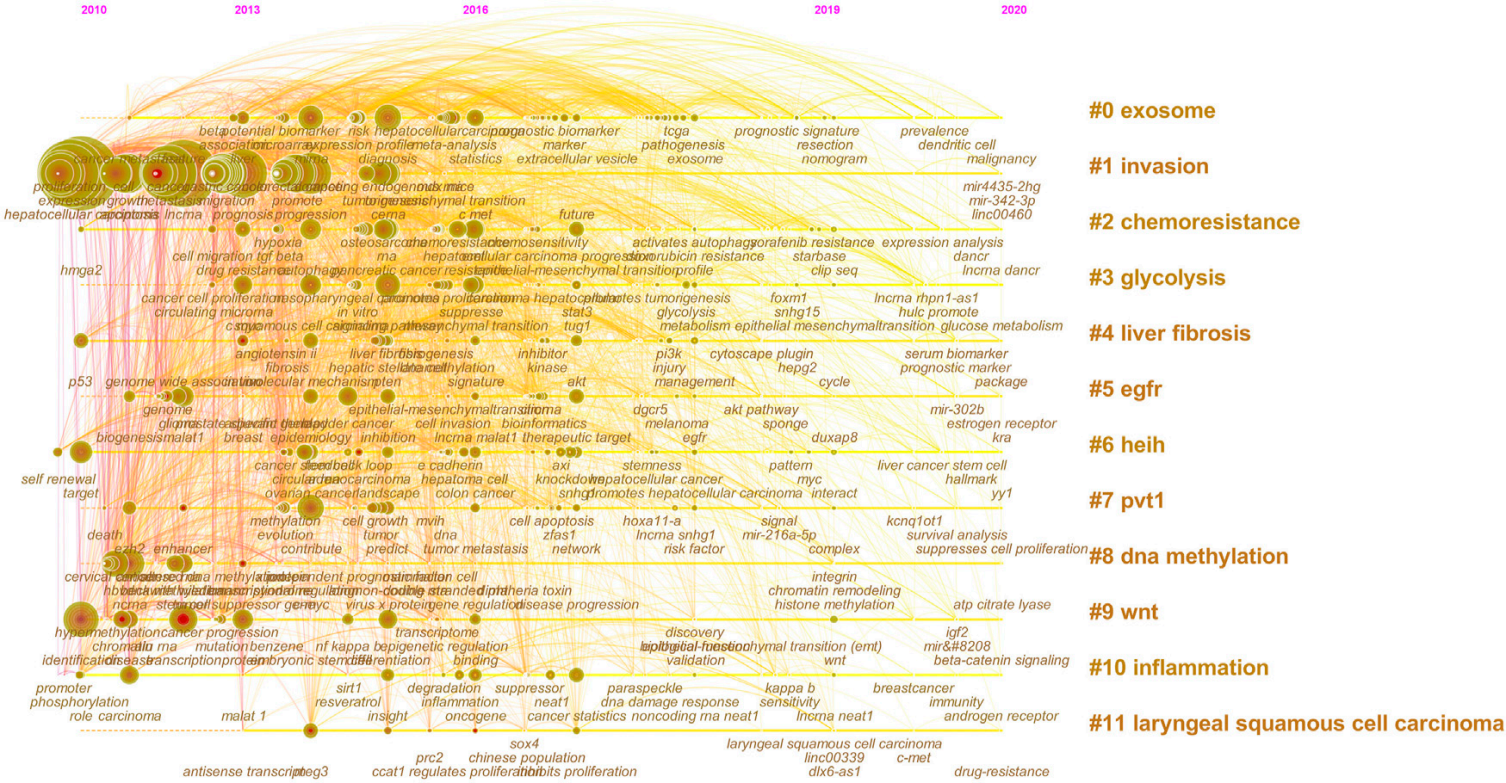
The timeline view of the knowledge map in lncRNAs and HCC.

In total, 780 research keywords were found in the field of lncRNAs in HCC development; only the keywords with a frequency value larger than 260 are displayed in Figure 6. The top 20 cited keywords are shown in Table 4, including hepatocellular carcinoma, lncRNA, expression, and so on. These words were classified into 11 large clusters (Figure 7): “exosome,” “invasion,” “chemoresistance,” “glycolysis,” “liver fibrosis,” “egfr,” “heih,” “pvt1,” “dna methylation,” “wnt,” “inflammation,” and “laryngeal squamous cell carcinoma.” This timeline view (Figure 7) visualized the most important keywords in a specific field and showed the emergence, popularity, and decline of the research topic. Burst keywords were regarded as an indicator for the frontiers of the specific field during a period of time. The CiteSpace version 5.6.R4 was used to explore the keywords with the strongest citation bursts (Supplementary Figure 1), and 86 keywords were detected. By combining the timeline view with the keyword burst map, we found the evolutionary path of research hotspots. At the earlier exploration stage (2010–2013), some rising terms were identified with identification, promoter, chromatin, expression, enhancer, etc. However, at the rapid development stage (2014–2018), some new terms with association to biological functions and diseases emerged, such as DNA methylation, induction, suppression, gene regulation, oncogene, liver cancer, tumor initiating cell, ovarian cancer, therapeutic target, etc. At the stable-growth stage (2019–2020), some emerging topics were observed, such as interact, wnt, starbase, and myc.

**TABLE 4:** Highly frequent Terms on the roles of lncRNAs in HCC development research.

Rank	Keyword	Freq
1	Hepatocellular carcinoma	2,032
2	lncRNA	1,589
3	Expression	1,189
4	Proliferation	1,035
5	Metastasis	815
6	Cancer	752
7	Prognosis	703
8	Invasion	542
9	Progression	465
10	Apoptosis	330
11	Migration	318
12	Gastric cancer	317
13	Growth	299
14	Promote	291
15	Gene	281
16	Biomarker	269
17	Lung cancer	260
18	Cell	258
19	Microrna	252
20	Colorectal cancer	252

### Analysis of Co-Cited References

Some papers which had been cited with a high frequency are listed in Table 5. “Yuan JH, DOI 10.1016/j.ccr.2014.03.010,” “Torre LA, DOI 10.3322/caac.21262,” and “Gupta RA, DOI 10.1038/nature08975” were the top three cited papers with the highest frequency. The total numbers of citations of the three aforementioned papers were 339, 321, and 258, respectively. These three cited papers were the fundamental cornerstone for this field.

**TABLE 5:** Highly frequent cited papers on the roles of lncRNAs in HCC development.

Rank	First author	Journal	IF (2020)	DOI	Total number of citations
1	Yuan JH	Cancer Cell	31.743	10.1016/j.ccr. 2014.03.010	339
2	Gupta RA	Nature	49.962	10.1038/nature08975	321
3	Torre LA	CA-Cancer J. Clin	508.702	10.3322/caac.21262	258
4	Bray F	CA-Cancer J. Clin	508.702	10.3322/caac.21492	222
5	Schmitt AM	Cancer Cell	31.743	10.1016/j.ccell. 2016.03.010	219
6	Yang F	Hepatology	17.425	10.1002/hep.24563	211
7	Salmena L	Cell	41.582	10.1016/j.cell. 2011.07.014	176
8	Wang KC	Molecular Cell	17.97	10.1016/j.molcel. 2011.08.018	172
9	Ponting CP	Cell	41.582	10.1016/j.cell. 2009.02.006	164
10	Yang Z	Annals of Surgical Oncology	5.344	10.1245/s10434-011-1581-y	162

## Discussion

To the best of our knowledge, this was the first study to explore the mapping intellectual structure for lncRNAs and HCC development by using CiteSpace. The number of published papers related to the field of lncRNAs in HCC development increased sharply in recent years. We found the knowledge base for the field of lncRNAs and HCC development by combining co-citation analysis with co-word analysis.

Regarding the top 10 prolific countries, four were developing countries, and six were developed countries. In terms of absolute publication numbers, China was the leading country, followed by the United States; this indicated that China had made significant progress in this area. However, China had a lower centrality value compared to the United States; it suggested that although China was the most prolific country in producing research publications, the United States played a leading role in collaborative network in this research field. There were extensive collaborations between western countries in the collaboration network. Transatlantic collaborations were the strongest among the United States, China, Germany, and Italy. The top 10 institutions were all from China. Among them, the papers published by Nanjing Medical University were cited with the highest frequency. For example, a study showed that Lnc-EGFR could suppress cytotoxic T lymphocyte (CTL) activity, stimulates Treg differentiation, and promotes HCC development through an EGFR-dependent signaling pathway (Jiang et al., 2017). Moreover, Zhuo et al. suggested that MEG3 serves as a prognostic biomarker for HCC (Zhuo et al., 2016). In addition to that, a previous study suggested that lncRNA ANRIL, as a cell growth modulator, may act as a novel potential therapeutic target for HCC (Huang et al., 2015).

In terms of the top 10 co-cited journals, six of the journals were from the United States. It was noteworthy that none of the top 10 co-cited journals were from China. However, Oncotarget was dropped by Science Citation Index Expanded. This demonstrated that these papers published in the journal need to be further improved. Moreover, impact factor ranged from 3.24 to 508.702. Additionally, this field had been more widely studied, and it covered more scientific disciplines in recent years, such as oncology (Lai et al., 2019; Lim et al., 2019; Wei et al., 2019), cell biology (Li et al., 2019; Song et al., 2019), and biochemistry molecular biology (Du et al., 2019; Guo et al., 2019). In the dual map, papers published in the molecular/biology/genetics journals were mostly cited by the studies published in molecular/biology/immunology and medicine/medical/clinical journals.

As for the top 10 prolific authors, each of them contributed to at least 11 publications. However, only one of the top 10 prolific authors was included in the top 10 co-cited authors, indicating that the authors should consider improving the quality of their articles in future. Co-cited authors include Yang F, who suggested that lncRNA-HEIH served as an oncogenic lncRNA that promotes tumor progression in HCC (Yang et al., 2011); Gupta RA, who indicated that lncRNAs played a positive role in regulating the cancer epigenome and could be the important targets in the diagnosis and therapy of HCC (Gupta et al., 2010); Torre LA, who reported that a large proportion of HCC cases and deaths could be effectively avoided by averting with appropriate preventive measures, such as vaccination, the use of early detection tests, and tobacco control (Torre et al., 2015); and Ponting CP, who explored the evolution of lncRNAs contributions to epigenetic gene regulation and transcriptional regulation (Ponting et al., 2009). Even though these authors were not prolific authors, they had already made significant contributions in this field.

Burst keywords were considered to discover emerging trends or research frontiers during a period of time (Chen, 2006). In this study, CiteSpace 5.6.R4 was used to capture the strongest citation bursts. At exploration stage (2010–2013), since the lncRNA HOTAIR had been proven to promote breast cancer metastasis by participating in chromatin remodeling (Gupta et al., 2010), researchers paid a lot of rising attention on lncRNAs. More and more functions of lncRNAs had been found. For example, lncRNAs could influence the development or progression of cancer (Ling et al., 2018; Chen and Xia, 2019). Subsequently, both the primary and secondary structures of lncRNAs had been discovered by enzymatic probing (Ilik et al., 2013) or chemical probing (Novikova et al., 2013; Smola et al., 2016). Researchers went one step further to divide lncRNAs into intronic, intergenic, sense, antisense, and bidirectional according to their genomic location relative to protein coding genes (Batagov et al., 2013).

At the rapid development stage (2014–2018), the mechanism, function, and application of lncRNA had been further studied. Based on the different mechanisms of lncRNAs acting on cellular processes, they are divided into five categories, such as signal (Pandey et al., 2008; Wang and Chang, 2011), decoy (Hung et al., 2011), guide (Wang and Chang, 2011), scaffold (Wang and Chang, 2011), and enhancer (Goyal et al., 2018; Liao et al., 2018). Moreover, some existing studies have suggested that the various functions of lncRNAs serve as critical roles in diverse biological processes, including epigenetic modification (Bhan and Mandal, 2014), transcriptional regulation (Wei et al., 2019), post-transcriptional regulation (Cesana et al., 2011), translational regulation (Yoon et al., 2012), and post-translational regulation (Wang et al., 2014). In addition, there had been more and more applications of lncRNAs discovered in cancer research. Initially, lncRNAs were reported to be associated with breast cancer (Shore et al., 2012). Some subsequent research were also reported in HCC (Shi et al., 2019), ovarian cancer (Oncul et al., 2020), and colorectal cancer (Huang et al., 2017). These lncRNAs could be used to work as novel prognostic biomarkers and intervention strategies for HCC.

At the stable-growth stage (2019–2020), with the rapid development of network public databases, there were many lncRNAs databases established, such as LNCipedia, LNCBook, TargetScan, Starbase (ENCORI), etc. Some of them were used to explore interactions (RNA–RNA interactions, RNA–DNA interactions, and RNA–Protein interactions) (Li et al., 2014) and pathway analyses (Qian et al., 2020), which were considered as a research frontier in the field of lncRNAs and HCC development. Furthermore, The Cancer Genome Atlas (TCGA) and Gene Expression Omnibus (GEO) databases could allow researchers to obtain raw data for free. These two databases were combined with some predictive or prognostic databases to perform bioinformatics analysis, such as UALCAN (Chandrashekar et al., 2017) and Kaplan–Meier Plotter (Hou et al., 2017), etc.

### Strengths and Limitations

This was the first study to address the roles of lncRNAs in HCC development research with CiteSpace. However, some study limitations should not be overlooked. First, this study did not include all relevant literatures. Even though majority of the research papers in the field of lncRNAs and HCC were included in the WoSCC database, some other databases such as Pubmed and Scopus may provide a broader scope. Second, there were discrepancies between the bibliometric analysis results and the actual research conditions due to a lower citation count from the recently published studies. Third, although all the data extraction was included within 1 day to avoid bias, the database is still expanding due to the daily updates of databases. Despite this, the vast majority of studies were included in this study. Therefore, the conclusion may not be impacted with a small amount of recent papers updated.

## Conclusion

This analysis urges other researchers to discover the research hotspots and frontiers in the field of lncRNAs and HCC development from year 2010 to year 2020. A total of 2,667 records on this topic were initially found worldwide. The annual related publications output had increased dramatically over this time. Although China was the most prolific country in terms of research publication, the United States played a leading role in collaborative network. The Nanjing Medical University was the most productive institute in this field. Gang Chen was the most prolific researcher, while Yang F was the most frequently co-cited author. Oncotarget, Cell, and Oncogene were the most highly co-cited journals. The most recent burst keywords were interaction, database, and pathway.

## Data Availability

The datasets presented in this study can be found in online repositories https://www.webofscience.com/wos/woscc/basic-search. The names of the repository/repositories and accession number(s) can be found in the article/Supplementary Material.
