# Supplementary figures and images for: Mapping Intellectual Structure for the Long Non-Coding RNA in Hepatocellular Carcinoma Development Research
Source: Front Genet. 2022 Jan 3;12:771810. doi: 10.3389/fgene.2021.771810 (PMC8762053; doi:10.3389/fgene.2021.771810)

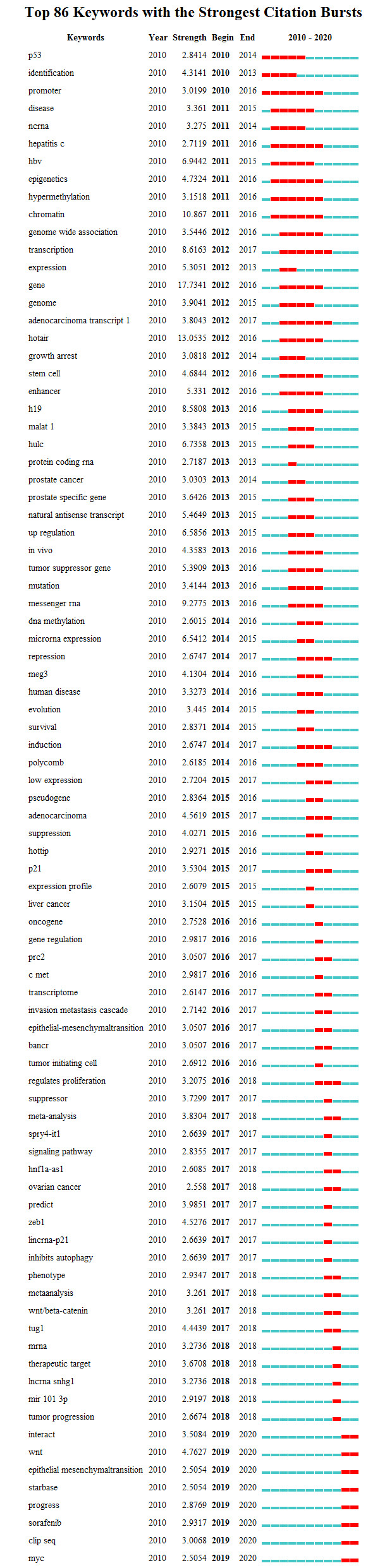

Supplement: Supplementary file 1 [file Image1.JPEG]
